# Ethics, emergencies and Ebola clinical trials: the role of governments and communities in offshored research

**DOI:** 10.11694/pamj.supp.2015.22.1.6216

**Published:** 2015-10-10

**Authors:** Morenike Oluwatoyin Folayan, Kristin Peterson, Frances Kombe

**Affiliations:** 1 Obafemi Awolowo University, Ile-Ife, Osun State, Nigeria; 2 Department of Anthropology, University of California, Irvine, USA; 3 KEMRI Wellcome Trust Research Programme, Kenya

**Keywords:** Ethics, Ebola, government, community engagement

## Abstract

The Ebola Virus Disease (EVD) in West Africa has stimulated investments in EVD research. While these research efforts are most welcome, we are concerned about the potential to ignore effective community ethics engagement programmes and critical government regulatory agencies in light of the urgency to conduct clinical trials for EVD therapies and vaccines. We discuss the reasons why community engagement with various research stakeholders is essential, how community engagement should be conducted, and the potential consequences of failing to engage both communities and regulatory agencies by drawing on past experiences in the field of HIV research. We highlight the importance of a) capacity building to enable local researchers design and implement EVD research for future epidemics, b) the need to support community research literacy, and c) the need to build the competency of research regulatory agencies on the continent to address EVD therapy and vaccine research.

## Introduction

The tenofovir controversies that erupted in the early 2000s resulted largely from differences in communities’ and researchers’ perspectives on what made an HIV prevention pre-exposure prophylaxis trial ethical. This was defined as ethical misrecognition by Peterson, Ukpong and colleagues [1–6] in their public discussions of these debates. When not addressed, such widespread concerns spanning continents were strong enough to result in the disruption and closure of most of the early PrEP trials [7].

Poor community engagement with Ebola Virus Disease (EVD) research may once again result in differing perspectives about research ethics. The current Ebola outbreak has resulted in over 8,000 deaths and over 20,000 infections [8] in a space of approximately 13 months following the first reported case in December 2013 [9]. Global concerns [10], fear of the infection spreading beyond Africa [11, 12] and the possibility of EVD being used for bioterrorism [13] have prompted large financial investments in EVD research. Due to the urgency to curb the EVD crisis, there are plans over the next two years to conduct eight clinical trials on Ebola treatment and eleven clinical trials on Ebola vaccines [14]. However, the timelines are so short that the prospect for effective community engagement is dismally low despite the now strong recognition to effectively engage local communities in the clinical research process [15]. The papers discusses the reasons for and how to engaged communities – identified in this discussion paper as stakeholders invested in EVD research conduct - in EVD research.

## Community engagement and EVD research

Community engagement would require extensive dialogue with key stakeholders, which should begin long before the trials are implemented. These include discussions with community members and community leaders at prospective trial sites on issues such as the study design, how the therapies or vaccines are imagined to work, plans and timelines for study implementation, the potential risks trial participants could encounter, the rights of trial participants, and how state authorities are involved in the design and implementation of trials in ways that protect the rights of study participants.

This form of community dialogue gives communities the opportunity to share perspectives on study designs, which helps reduce challenges that may be associated with study participants’ recruitment and retention [16]. The process also facilitates community ownership of research processes and research outcomes thereby expediting translation of research findings into use - a common challenge with many research endeavours [17]. Community dialogue also promotes understanding of research concepts, reduces therapeutic misconceptions and myths about the research [18, 19]. They also strengthen the informed consent process through dissemination of information on research goals, risks and benefits; they also help to instill respect of social norms and practices of potential volunteers [20, 21].

In some cases, community dialogues are needed to negotiate and reach consensus on standard of prevention and care packages for study participants as these may differ between researchers and trial volunteers [22]. When community dialogues are conducted, researchers should expect that components of the study design may be deemed unacceptable or unethical by community members. Researchers should be prepared to negotiate and rectify any potential disagreements that dissatisfy community members. One of the main reasons why the early tenofovir trials were shut down was because such disagreements between researchers and potential were never resolved. This process of active community engagement before, during and after research promotes respect for the community and strengthens trust and credibility of researchers [20, 23]. It also gives the community a sense of ownership of the research and increases their interest in the research process [20].

The feeling of clinical trial exploitation in the region [24] has a long history. Community members in Nigeria, for example, have raised concerns about unethical practices conducted by offshored research [25]. While we do not believe that community engagement may altogether address the concerns raised, nor that community engagement is the only panacea needed for hitch free clinical trials. It, however, goes a very long way in preventing many of the potential problems that could otherwise have resulted without credible community engagement programmes.

Other stakeholders that need to be engaged during EVD research include national governments. All current EVD therapeutic and vaccine research is initiated by partners from the global North. Research specimens may need to be exported from trial sites to institutions in the global North for investigational analysis not feasible in many of the research communities due to the lack of infrastructure to conduct such levels of molecular analysis. This process has the potential of creating a lot of suspicion and mistrust if not well handled and documented. Ideally, such exportation of specimens requires the signing of material transfer agreements between researchers and the government of the host country through appropriate agencies. In Nigeria, the National Health Research Ethics Committee hosted by the Federal Ministry of Health plays this role [26]. The Terms of Reference must be developed transparently and clearly spelled out with mandates for all parties, including access agreements and intellectual property rights. Moreover, the current sidelining of African research scientists in North-South global health collaborations is quite rampant and may become a serious issue and concern. Effort to create transparent multiple author publishing agreements is important in this regard.

The engagement of the national government also helps facilitate discussions about technology transfers and future access to vaccines and therapies. Technology transfer to countries where EVD research would be conducted is crucial as the competency for clinical trial conduct in these countries is low [27, 28]. There is little possibility of researchers receiving funds and infrastructure from the South in order to initiate EVD research studies independent of collaborations with Northern partners. Without concerted efforts to ensure that current investments in EVD research results in technology transfers and capacity building, it is likely that locally initiated researches to address future EVD outbreaks would be very few.

Government's negotiation with research sponsors is also critical to ensure future access to developed therapies when needed at affordable prices [29]. Such negotiations are appropriately done during the study design stage with signing of memorandums of understanding between both parties. Unfortunately, current discussions on future access to EVD therapies have been limited [30, 31].

Also, we are concerned about how regulatory authorities in these countries would handle reviews, approval, and monitoring of study protocols. Affected countries could be very easily overwhelmed with the need to review clinical trial protocols for which they do not have local competency to provide review. Many ethics committees in Africa review research protocols to ensure their scientific validity and ethical integrity. In addition, a number of research ethics committees need to provide monitoring oversight [32]. Yet, the human resources to provide scientific and ethical review as well as monitoring of these complex clinical trials, associated with various ethical dilemmas, are largely non-existent [33]. Worse still, the competency to provide regulatory oversight for clinical trials is less than optimal in many African countries including those proposed for EVD research. Many lacked the necessary expertise to conduct the activities involved in the regulation of clinical trials [34]. Concerns have therefore been raised about the possibility of arising unethical practices like the case of the Pfizer's Trovan trial during a meningitis outbreak in Nigeria [35]. Indeed, this trial was very quickly designed and implemented with no Nigerian ethical clearance and ultimately disastrous results - many children trial participants were maimed or died. Similar quick designed and fast-paced EVD trials are currently being touted as a favorable approach to EVD research. Indeed, communities in the recent past have shared many concerns about little or poor adherence to the regulations guiding clinical trials instituted by overseas science communities [34]. Therefore the need for effective state monitoring of EVD related clinical trials and other forms of research, is essential.

In recognition of this gap, the ninth African Vaccine Regulatory Forum was held in November 2014 in order to agree on a collaborative mechanism for fast tracking clinical trials approvals and registration of these products in the affected countries [36]. One of the outcomes of the meeting was the recognition that there is need to provide support and expertise to help with the reviews of application submissions for ethical review licensure of EVD vaccines. The need for capacity building for research regulatory agencies in the EVD affected region was also identified [37].

## Conclusion

While we applaud the support for research into Ebola, we believe that a comprehensive research plan needs to be developed for the EVD research process. Countries new to such level of clinical research should not be unduly burdened with handling significant new issues that govern the conduct of research. South-South collaboration should be encouraged to facilitate mentorship and capacity building processes during EVD research as exemplified by the Lao-Thai-Australia collaboration in HIV nutrition [38].

It is essential that the current EVD research process builds local research and community capacity in ways that ensure a great deal more capacity is left behind in the countries where research takes place. Support should be provided to institutions that host EVD related researches in the EVD region to enable them build research infrastructure; local researchers should be engaged during the development of research protocols so that they can lay claims to authorship rights and research outcomes; local researchers’ participation in EVD research should go beyond being data collectors and co-authors on published papers; participation should be driven by a desire to ensure and support future local initiatives for EVD research in the region. Community engagement should be seen as not only an ethical imperative but a necessity that helps to avoid problems and conflicts that lead to premature trial closures. In the short term, lay persons on ethics committees can be trained to review and address community issues in research protocols [39, 40] or/and be supported to establish independent community advisory boards. This should not preclude community engagement efforts that promote research literacy, community dialogue, and the building of community trust. These efforts are essential in reducing the chances that the current donor supported EVD research in Africa be perceived as exploitative in the future.
